# Infection of human monocytes by *Chlamydia pneumoniae* and *Chlamydia trachomatis*: an *in vitro* comparative study

**DOI:** 10.1186/1756-0500-7-230

**Published:** 2014-04-11

**Authors:** Antonella Marangoni, Christian Bergamini, Romana Fato, Claudia Cavallini, Manuela Donati, Paola Nardini, Claudio Foschi, Roberto Cevenini

**Affiliations:** 1 Microbiology, DIMES, University of Bologna, S.Orsola Hospital, via Massarenti 9, 40138 Bologna, Italy; 2 FaBiT Department, University of Bologna, Bologna, Italy; 3 Laboratory of Molecular Biology and Stem Cell Engineering, DIMES, University of Bologna, Bologna, Italy

**Keywords:** Human monocytes, *Chlamydia pneumoniae*, *Chlamydia trachomatis*, Reactive oxygen species, Reactive nitrogen species, Cytokines

## Abstract

**Background:**

An increasing number of studies suggest that chlamydiae can infect immune cells. The altered immune cell function could contribute to the progression of several chronic inflammatory diseases.

The aim of this study was to comparatively evaluate *Chlamydia pneumoniae* (CP) and *Chlamydia trachomatis* (CT) interactions with *in vitro* infected human blood monocytes.

**Results:**

Fresh isolated monocytes were infected with viable CP and CT elementary bodies and infectivity was evaluated by recultivating disrupted monocytes in permissive epithelial cells.

The production of reactive oxygen and nitrogen species was studied in the presence of specific fluorescent probes. Moreover, TNF-α, INF-α, INF-β and INF-γ gene expression was determined.

CT clearance from monocytes was complete at any time points after infection, while CP was able to survive up to 48 hours after infection. When NADPH oxydase or nitric oxide synthase inhibitors were used, CT infectivity in monocytes was restored, even if at low level, and CT recovery’s rate was comparable to CP one.

CT-infected monocytes produced significantly higher levels of reactive species compared with CP-infected monocytes, at very early time points after infection. In the same meanwhile, TNF-α and INF-γ gene expression was significantly increased in CT-infected monocytes.

**Conclusions:**

Our data confirm that CP, but not CT, is able to survive in infected monocytes up to 48 hours post-infection. The delay in reactive species and cytokines production by CP-infected monocytes seems to be crucial for CP survival.

## Background

Chlamydiae are obligate intracellular bacteria that primarily infect mucosal epithelial cells at the sites of entry to the human body. Depending on the serovar, human infection with *Chlamydia trachomatis* (CT) causes a variety of ocular and genital diseases [1,2].

*C. pneumoniae* (CP) is involved in respiratory infections, mainly in community-acquired pneumonia, and there is some evidence of its implication in chronic conditions such as atherosclerosis and chronic obstructive pulmonary disease and/or asthma [3,4].

An increasing number of in vitro studies suggest that both CT and CP can be isolated from human peripheral blood and can infect immune cells, at least at a low level, [5,6]. Furthermore, several differences have been reported, depending on the origin of immune cells used (i.e., animal vs. human cells, residential immune cells vs. circulating cells, cell lines vs. freshly isolated cells), chlamydial MOI (multiplicity of infection) used or CT genotypes [7]–[12].

Changes in the cellular redox state can trigger mechanisms that are crucial for the cell life: reactive oxygen species (ROS) production - in the early stages of a microbial infection - is a valuable defense mechanism used to kill the infecting agent [13]. ROS are also involved in the mechanisms of replication and cell death. While low levels of these substances indeed stimulate cell proliferation, high ROS levels induce cell death. There is evidence from literature that the survival of an infectious agent within a host cell is linked to its ability to induce a state of oxidative stress, while a reduced ROS production promotes the establishment of a chronic infection [14]. The intracellular ROS level is the result of a continuous balance between the production and the antioxidant defenses [15]. An increase in ROS production or a decrease in antioxidant defenses leads to an imbalance of the redox state, which can result in apoptosis [16]. Azenabor and his collaborators [17] showed that macrophages infected by CP produced ROS through membrane-associated NADPH oxidase with oxidative phosphorylation levels depending on Ca^2+^ influx signals.

The same authors recently [18,19] found a Ca^2+^-signal mediated anti-inflammatory response in CT- infected macrophages. Moreover, they were able to demonstrate that, in macrophages infected with CP, the activities of antioxidant enzymes such as SOD and GPx increased within the first few hours after infection [20]. It is nevertheless noteworthy to underline that all the results obtained by this group derived from their infection data on THP-1 cell line rather than primary cells, such as freshly isolated human monocytes or animal peritoneum-derived macrophages.

Taken together, these findings show that chlamydial replication in monocytes/macrophages is limited and that infection of immune cells results in the secretion of a range of cytokines, production of reactive species, resistance to apoptosis and alteration of different macrophage functions.

The aim of the present work was to evaluate infection characteristics, ROS and reactive nitrogen species (RNS) production and cytokines gene expression in a comparative model of isolated monocytes infected by two different *Chlamydia* species (CP and CT). Our study could help to shed light on chlamydial differences, which might account for the different biological behaviors of these pathogens, as well as the clinical outcome of the diseases linked to their infections.

## Methods

### Isolation of monocytes

Blood was collected from ten healthy volunteers and a written informed consent was obtained from all the subjects. The study protocol was reviewed by the institutional Ethics committee at St. Orsola Hospital. The volunteers were screened for excluding the presence of antibodies against genus-specific *Chlamydia* antigens. Only seronegative subjects, with no history of CT genitourinary or CP respiratory infections were admitted to the study. Peripheral blood mononuclear cells (PBMCs) were isolated by centrifugation over Ficoll-Paque (GE Healthcare Bio-Sciences AB, Uppsala, Sweden) at 400 × *g* for 30 minutes. Monocytes were subsequently separated from PBMCs by centrifugation and adherence for 1 h [10]. Isolated monocytes were washed thoroughly with RPMI 1640 and cultured in RPMI 1640 with 10% fetal calf serum (FCS), 2 mM L-glutamine, 100 units/ml penicillin, and 100 μl/ml streptomycin, and maintained overnight at 37°C in 5% CO_2_. The day after the isolation, monocytes were gently washed with 10% FCS-RPMI 1640. Cell viability was checked using the trypan blue exclusion method (viability >99%). Cell purity was checked by performing immunofluorescence with a monoclonal mouse anti-human CD14 antibody (Abcam, Cambridge, UK) and a fluorescein-conjugated anti-mouse antibody (Dako, Glostrup, Denmark); more than 98% of the cells were CD14 positive.

### Chlamydial organisms culture and purification

Chlamydiae used in this study were FB/96 strain of *C. pneumoniae* (CP) and *C. trachomatis* (CT) serotype D, strain GO/86. These two strains had been isolated from a patient with pneumonia and a patient with non-gonococcal urethritis, respectively, at St. Orsola Hospital of Bologna [21,22]. Chlamydiae were grown on confluent monolayers of epithelial LLC-MK2 cells (ATCC CCL-7), in Earle’s minimal essential medium (EMEM) with 1% (v⁄v) L-glutamine, 10% FCS, 1 μg/ml cicloheximide and 5 g/l glucose [23]. Chlamydial EBs were purified using renografin gradients, re-suspended in 0.2 M sucrose-phosphate-glutamic acid (SPG), and frozen in 0.5-ml aliquots at -80°C, as previously reported [24].

### Infection of monocytes and LLC-MK2 cells with viable CT and CP

Fresh isolated monocytes were seeded on glass coverslips in 24-well plates (5 × 10^4^ cells/well) and maintained overnight before infection. The cells were infected with viable CP and CT EBs in SPG buffer (infectivity ratio: 5 EBs/cell) for 60 min, washed twice with RPMI 1640, and then incubated in RPMI 1640, 10% FCS, 2 mM L-glutamine, 50 μg/ml vancomycin, and 10 μg/ml gentamycin, at 37°C in 5% CO_2_ for up to 96 h. At 24, 48, 72 and 96 hours post-infection, infected monocytes were ultrasonically disrupted and the supernatants were used to infect fresh monolayers of LLC-MK2 by centrifugation at 900 × *g* for 1 h, in order to evaluate the re-cultivation of viable EBs in epithelial cells, as already described [25]. Infection of LLC-MK2 cells by CT and CP was carried out as a positive control. At the same time points above indicated, LLC-MK2 cells infected by CT or CP were disrupted and the supernatants were used to infect fresh permissive monolayers cells. When required, infection of monocytes was carried out in presence of Apocynin 300 μM, a NADPH-oxydase inhibitor or L-NAME 300 μM, a nitric oxide synthase (NOS) inhibitor (Abcam, Cambridge, UK).

### Assessment of chlamydial inclusions

LLC-MK2 cells were fixed with methanol 3 days after the infection and stained with a fluorescein-labeled MAb to the LPS of *Chlamydia* (Meridian Diagnostics, Inc., Cincinnati, Ohio). The number of IFU/ml was determined by fluorescence microscopy as described elsewhere [26].

### Assay of ROS and RNS

After an overnight incubation at 37°C, monocytes seeded on 24-well plates (1 × 10^5^ cells/well) were infected with viable CT and CP EBs in SPG buffer (infectivity ratio: 5 EBs/cell) for 60 min in the presence of 10 μM Dichlorofluorescein diacetate (DCFDA) or 5 μM Diaminofluorescein (DAF-2), ROS and RNS probes, respectively. Treatments with 0.8 μM Phorbol or 10 μg/ml *Escherichia coli* Lipopolysaccharide (LPS) (Sigma-Aldrich, St. Louis, MO, USA) were used as positive controls to induce ROS or RNS production, respectively [27,28]. Cells were subsequently washed with phosphate buffered saline (PBS) and each well was filled with 300 μl of PBS and glucose (4,5 mg/ml). The increase in cell fluorescence from each well was measured (λexc = 485 nm; λem = 535 nm) with a spectrofluorometer (Wallac Victor multilabel counter, Perkin-Elmer Inc., Boston, MA, USA) at 3, 6 and 24 hours post infection.

### Real-time RT PCR for cytokines expression

After an overnight incubation at 37°C, monocytes seeded on 24-well plates (2 × 10^5^ cells/well) were infected with viable CP and CT EBs and (infectivity ratio: 5 EBs/cell) for 60 min; after two washing cycles with RPMI 1640, cells were incubated up to 24 hours. In each experiment, a positive control for cytokine production was included by stimulating monocytes with 10 μg/ml LPS (Sigma-Aldrich, St. Louis, MO, USA).

Cells were lysed in culture dishes by adding 1 ml of Trizol Reagent (Invitrogen, CA, USA). After incubation for 5 minutes at 25°C, 200 μl of chloroform was added. Sample was centrifuged at 12000 × g for 5 minutes and the upper aqueous phase transferred in a new tube. 3.5 volume of RLT buffer (Qiagen, GmbH, Hilden, Germany) and 2.5 volume of ethanol were added and the sample was applied to a Qiagen RNeasy micro column (Qiagen GmbH, Hilden, Germany). Total RNA was isolated following the manufacturer’s instructions. For RT-PCR, cDNA was synthesized in a 20 μl reaction volume with 500 ng of total RNA and SuperScript III RT (Invitrogen, CA, USA). Real-time RT-PCR was performed with 50 ng cDNA in a Lightcycler system (Roche Diagnostics, Mannheim, Germany), with the SYBR Green FastStart kit (Lightcycler® FastStart DNA Master^PLUS^ SYBR Green I, Roche Diagnostics, Mannheim, Germany). Primers used in real-time RT-PCR to assess TNF-α, INF-α, INF-β, INF-γ and GAPDH levels were from SuperArray (SABiosciences Corporation, Frederick, MD, USA). The reaction mixture (20 μl) contained 4 μl of Master SYBR Green I mix (*Taq* DNA polymerase, buffer, deoxynucleoside trisphosphate mix, MgCl_2_, and SYBR Green I dye) and 0.5 μM of each primer, to which 2 μl of cDNA was added. Data were normalized using GAPDH as an index of cDNA content after reverse transcription. Amplification included initial denaturation at 95°C for 10 min, 35 cycles of denaturation at 95°C for 10 s, annealing at 55–65°C for 6–10 s, and extension at 72°C for 10 s performed at a temperature transition rate of 20°C/s. Fluorescence was measured at the end of each extension step. Specificity of the product was determined by a melting curve analysis, conducted after completion of the cycling process with the aid of a temperature ramp (from 55°C to 95°C at 0.1°C/s) and continuous fluorescence monitoring. Samples were run in duplicate and the average threshold cycle (C_t_) value was used for calculations. REST^©^ (Relative Expression Software Tool, Qiagen GmbH, Hilden, Germany) software was used to analyze data. The mathematical model used was based on the correction for exact PCR efficiencies and the mean crossing point deviation between sample group(s) and control group(s). Expression ratio results of the investigated transcripts were subsequently tested for significance by a Pair Wise Fixed Reallocation Randomization Test^©^ and plotted using standard error (SE) of estimate via a complex Taylor algorithm.

### Statistical analysis

Data were analyzed by Student’s *t* test and P values <0.05 were reported as significant. For Real-Time RT PCR data, REST^©^ software was used. P values <0.05 were reported as significant.

## Results

### Recovery of viable chlamydiae from monocytes

**Figure 1:**
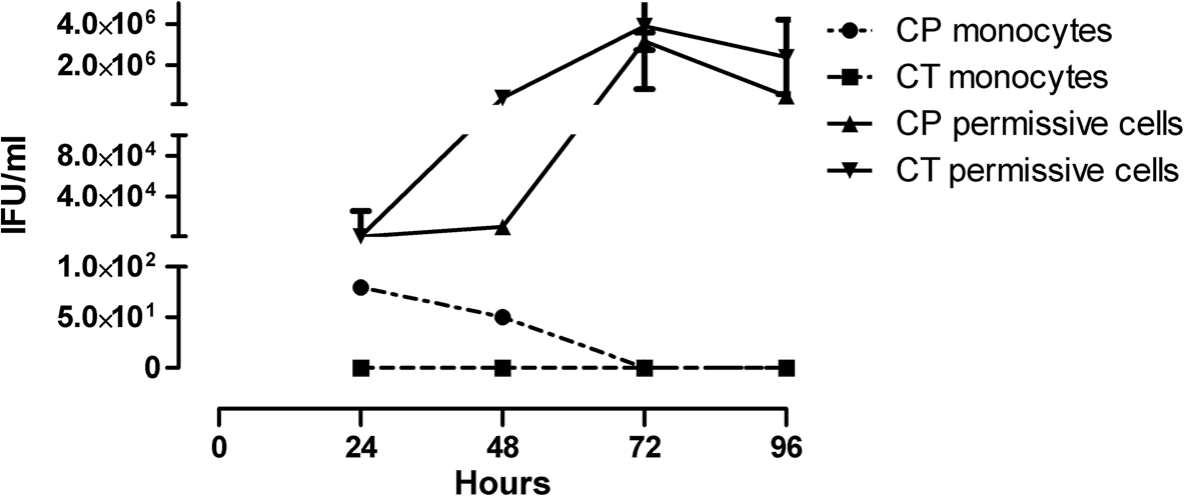
**Recovery of infectious chlamydiae after disruption of monocytes.** At 24, 48, 72 or 96 hours post-infection, monocytes or similarly infected permissive LLC-MK2 cells were disrupted and EBs infectivity was titrated in LLC-MK2 cells. At 3 days after the initial inoculum, chlamydial inclusions were enumerated. A representative from three independent experiments is shown (mean ± standard error of the mean-SEM).

A one-step growth curve for chlamydiae in monocytes and permissive LL-CMK2 cells was constructed (Figure 1). We were not able to recover infectious CT EBs from monocytes at any time after the infection, as already reported [8].

**Figure 2:**
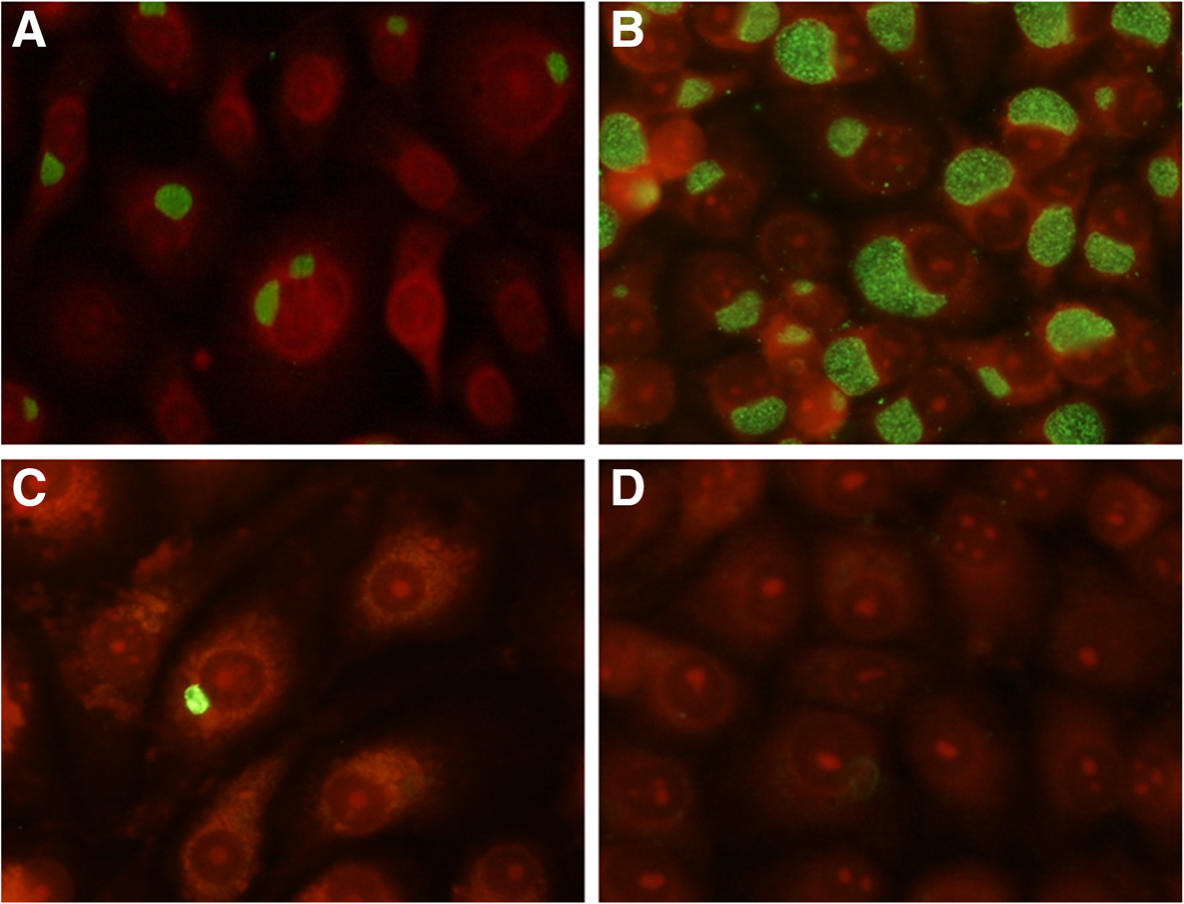
**Assessment of chlamydial inclusions by immunofluorescence.** Monocytes were infected by CP or CT in parallel with LLC-MK2 cells. At 24 hours post-infection, the cells were disrupted and the supernatants were used to infect fresh permissive monolayers cells. At 3 days after the inoculum, chlamydial inclusions were observed by fluorescence microscopy (400× magnification) after incubation with fluorescein-conjugated monoclonal antibodies against *Chlamydia* genus-specific LPS. **A**: LLC-MK2 inoculated with supernatant of CP-infected permissive cells. **B**: LLC-MK2 inoculated with supernatant of CT-infected permissive cells. **C**: LLC-MK2 inoculated with supernatant of CP-infected monocytes. **D**: LLC-MK2 inoculated with supernatant of CT-infected monocytes.

On the contrary, CP recovery was possible up to 48 hours post-infection, even if at low level, as already found by Wolf et al. [29]. Re-cultivation rate of CP differed significantly from CT at both 24 and 48 hours post-infection (P <0.01). An example of the infectivity assay results obtained in LLC-MK2 line is reported in Figure 2.

**Figure 3:**
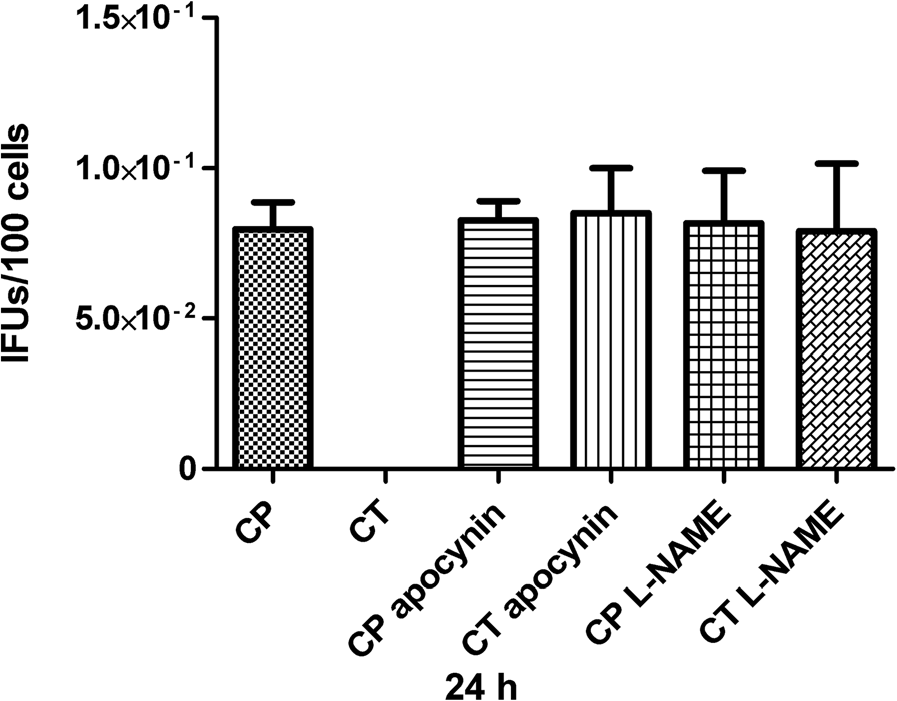
**Recovery of infectious chlamydiae after monocytes’ infection in presence of Apocynin or L-NAME.** CT or CP infection was carried on in presence of Apocynin 300 μM or L-NAME 300 μM. As control, monocytes infection was carried on with no NADPH-oxydase or NOS inhibitors. At 24 hours post-infection, monocytes were disrupted and EBs infectivity was titrated in LLC-MK2 cells. At 3 days after the initial inoculum, chlamydial inclusions were enumerated. Since at 24 hours post-infection the viability of uninfected monocytes in presence of Apocynin or L-NAME was less than 30%, and similar low viability values were observed in CT or CP infected cells, with no significant differences, here recultivation results are presented as IFUs/100 cells in order to normalize the data obtained. A representative from three independent experiments is shown (mean ± standard error of the mean-SEM).

Preliminary experiments performed in order to check uninfected monocytes’ viability in presence of Apocynin or L-NAME showed that at 24 hours post-infection less than 30% of the cells were alive, being the viability at 48, 72 and 96 hours after infection less than 15%. Therefore, chlamydiae recovery from inhibitors’ treated monocytes was evaluated at 24 hours post-infection. The viability of CP and CT infected cells was equal to 29.2% and 28.3%, respectively, when treated with Apocynin, whereas the viability values of CP and CT infected cells were 28.2% and 29.4%, respectively, for L-NAME treatment (P >0.05). As shown in Figure 3, CT recovery from Apocynin or L-NAME treated cells was possible, even if at low level. No significant differences were observed comparing CT to CP recovery results when Apocynin or L-NAME treated cells were evaluated (P >0.05).

### Reactive species production

Up to 6 hours after the infection, only CT-infected monocytes were able to produce ROS, when compared to untreated control (P < 0.05). The amount of RNS produced by CT-infected monocytes was significantly higher than both control and CP-infected cells up to 3 hours post-infection (P < 0.05). CP-infected monocytes did not show a significant reactive species production up to 6 hours post-infection, while a strong increase in both ROS and RNS production was observed at later time points, without differences between CP and CT-infected monocytes.

**Figure 4:**
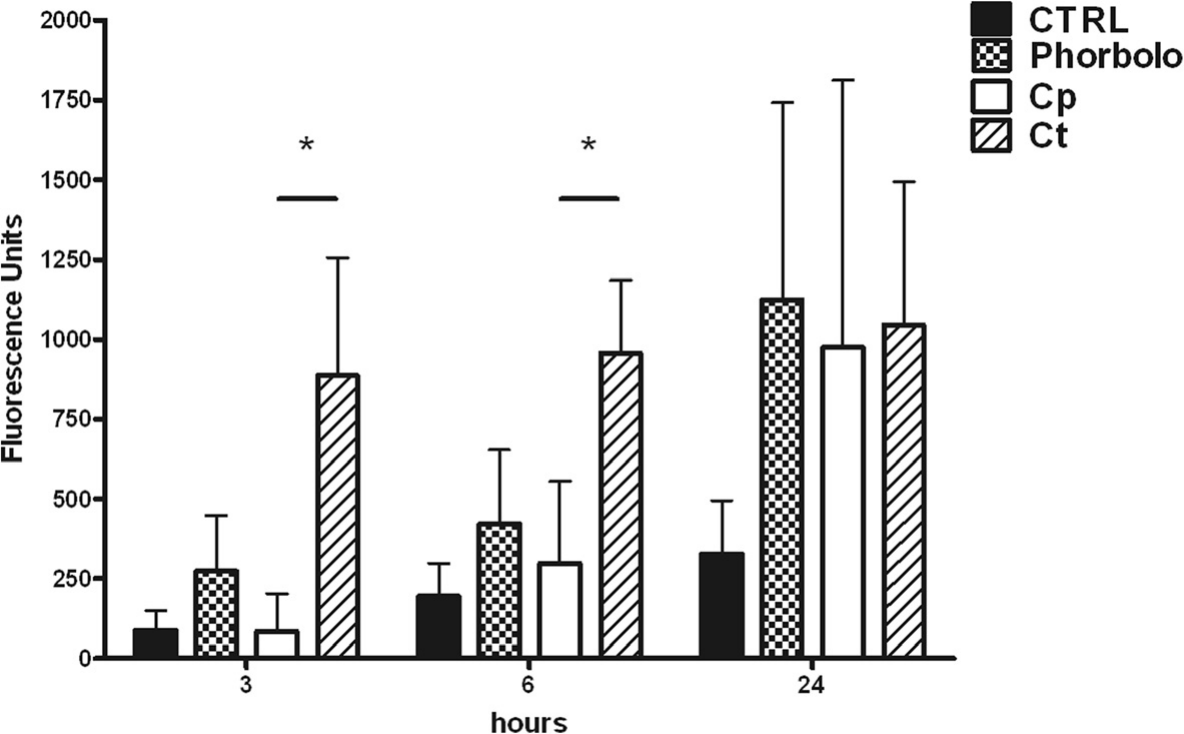
**ROS production.** Reactive Oxygen Species (ROS) production in human monocytes infected by CP or CT at 3, 6 and 24 hours post-infection, detected by means of the fluorogenic probe DCFDA. 0.8 μM Phorbol treatment was used as a positive control. (*P < 0.05; n = 4).

**Figure 5:**
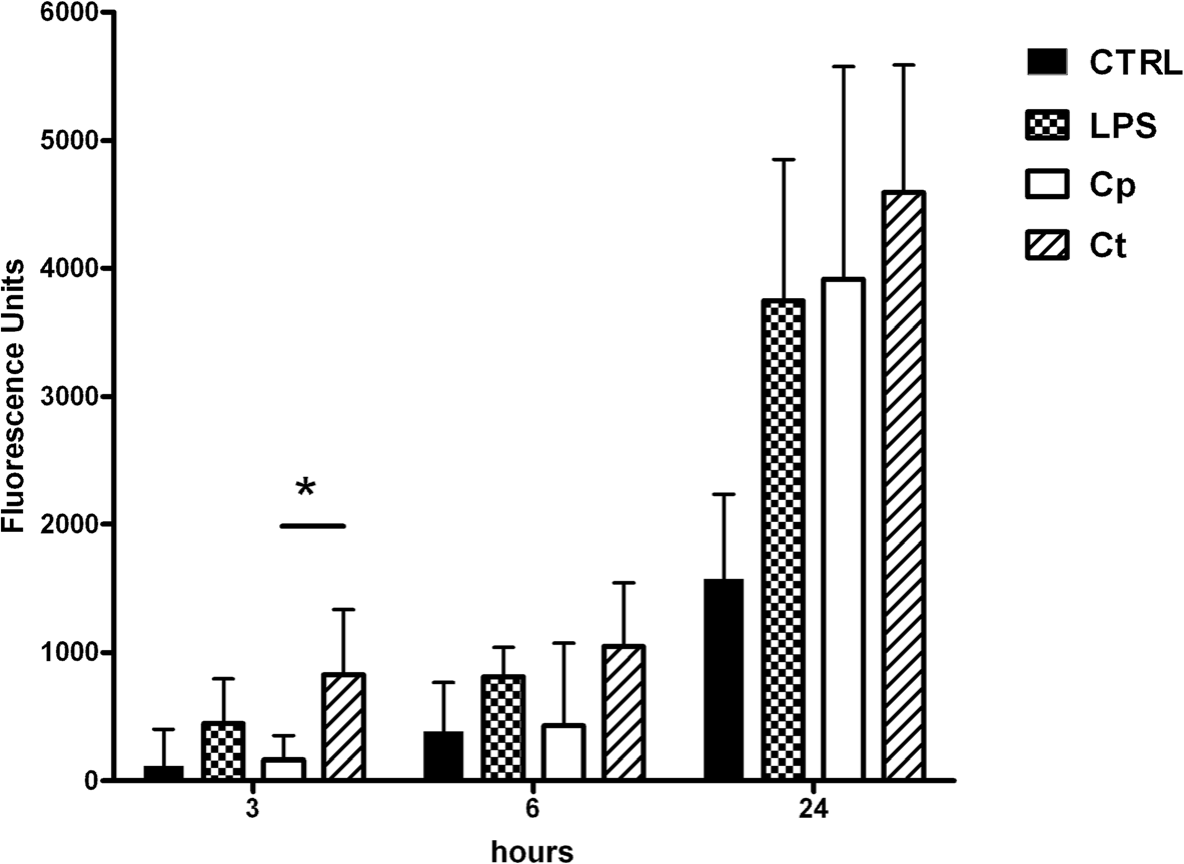
**RNS production.** Reactive Nitrogen Species (RNS) production in human monocytes infected by CP or CT at 3, 6 and 24 hours post-infection, detected by means of the fluorogenic probe DAF-2. 10 μg/ml LPS treatment was used as a positive control. (*P < 0.05; n = 4).

The time course of reactive oxygen and nitrogen species production in monocytes after the chlamydial infection is reported in Figures 4 and 5, respectively. Data were reported as mean ± standard deviation of four independent experiments.

### Real-Time RT PCR

Preliminary experiments conducted up to 24 hours showed that gene expression in CP and CT infected monocytes peaked at 3 hours, showing similar values to unstimulated controls at 12 and 24 hours post-infection. Consequently, all the further experiments considered only three time points, at 1.5, 3 and 6 hours post-infection, respectively.

**Figure 6:**
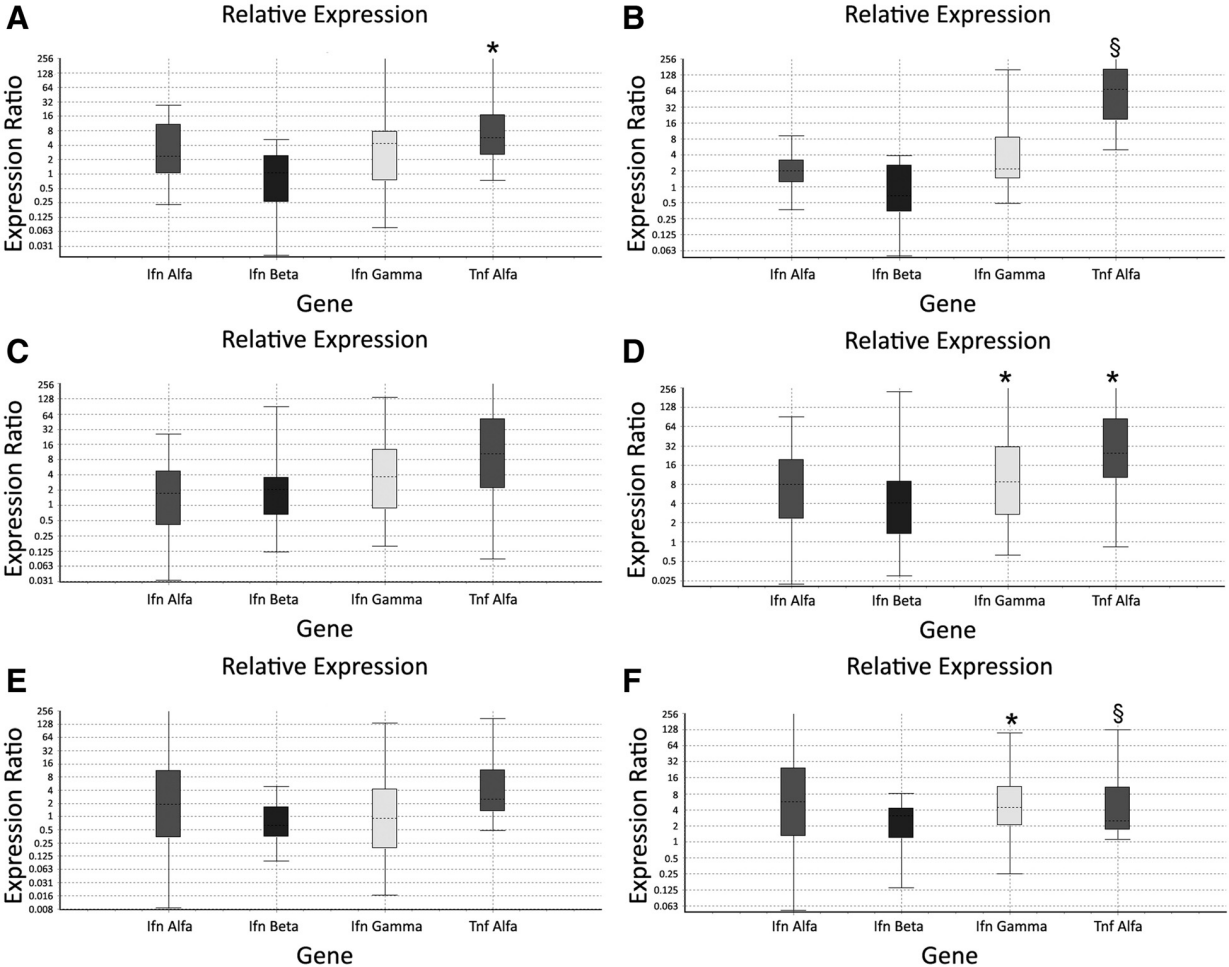
**Cytokines gene expressions.** Analysis of gene expressions. Fold increase in gene expression of INF-α, INF-β, INF-γ and TNF-α analyzed in CP-infected monocytes **(A, C, E)** and CT-infected monocytes **(B, D, F)** compared to controls (uninfected monocytes) at 1.5 **(A, B)**, 3 **(C, D)**, 6 **(E, F)** hours post-infection. *Significantly different from control group P < 0.05. ^§^Significantly different from control group P < 0.01.

Monocytes stimulated by CT showed a significantly higher TNF-α gene expression than uninfected cells (P < 0.01 at 1.5 and 6 hours, P < 0.05 at 3 hours). INF-α and INF-β gene expression was only slightly increased at 3 and 6 hours after the infection in CT-infected monocytes, compared to uninfected cells. On the other hand, at 3 and 6 hours post-infection INF-γ gene expression was significantly higher in CT-stimulated monocytes than in unstimulated cells (P < 0.05). Regarding CP-infection, the only significant difference compared to unstimulated cells was observed in TNF-α gene expression at 1.5 hours post-infection (P < 0.05). Anyway, TNF-α gene expression of CP-stimulated cells was significantly lower than the corresponding one observed in CT-infected monocytes (P < 0.05). Cytokines gene expression in the different models of infection is presented in Figure 6; the results are reported as fold increase in gene expression of at least three independent experiments.

## Discussion

The particular interaction between chlamydiae and immune cells has been widely investigated to define the role of these intracellular bacteria in human diseases such as atherosclerosis and coronary artery disease, asthma, Alzheimer’s disease, multiple sclerosis and reactive arthritis [30-32].

Due to the absolute requirement of a cellular host for the production of a new progeny, the gold standard to demonstrate chlamydial replication should involve isolation of infectious EBs and regrowth of chlamydiae in new epithelial cells. The LLC-MK2 cells, here used, demonstrated to be highly susceptible to chlamydiae in previous studies [24-26]; in particular, chlamydial inclusions are well detectable by fluorescence microscopy, when fluorescein-conjugated antibodies anti-*Chlamydia* are used.

Our in vitro data show that, in human monocytes, CT replication has been successfully cleared: infectious CT EBs were never recovered from infected monocytes at any time after the infection. In contrast, CP infectivity has been far reduced at 24 and 48 hours post-infection, but CP was still present in monocytes in its infective form. Only at later time points CP infectivity was totally prevented. In vivo, the presence of CP in infected monocytes could be the condition for the dissemination of this pathogen, via the blood stream, to different tissues [33,34]. The first 48 hours after infection are critical for its spread to more permissive tissues and can explain the recovery of CP in atherosclerotic lesions. As for monocytes activation, we tested reactive species production in freshly isolated monocytes infected by CT and CP EBs, using DCFDA and DAF as fluorescent probes for ROS and RNS, respectively. We found major differences between the two species: in particular, at early time points after the infection, only CT was able to elicit an important production of ROS and RNS. In contrast, at 24 hours, both CT and CP-infected monocytes showed a sharply increased production of reactive species.

While the central role of ROS in chlamydial infections has already been underlined [20,35], the role of nitrogen reactive species still needs to be elucidated. In this connection, Ramsey reported that inducible nitric oxide synthase (i-NOS)-deficient mice sustain a significantly increased rate of disease than non-deficient animals in CT genital infections [36], whereas Rothfuchs [37] demonstrated that i-NOS was necessary for effective CP clearance by murine bone marrow-derived macrophages. In the present study, the use of a NADPH-oxydase inhibitor or a NOS inhibitor was essential to sustain CT infectivity in human monocytes, at least at low level, showing that reactive species are strongly involved in CT clearance from infected cells.

Unfortunately, these inhibitors highly reduced monocytes viability, so in our model we were not able to evaluate reactive species production or cytokines gene expression in the presence of Apocynin or L-NAME, since the results would have been highly affected by monocytes’ lack of viability. Further studies with other compounds or different culture conditions could contribute to answer the unsolved questions.

It is well established that nitric oxide produced after cell activation by INF-γ is important for killing or inhibiting growth of different microorganisms [38,39].

In this context, it is noteworthy to underline that we found a striking feature of CT-induced INF-γ expression, compared to CP-infected cells. Moreover, also TNF-α gene expression was significantly higher in CT-infected cells than in both CP-stimulated or unstimulated cells.

There is strong evidence that monocytic cells infected by several *Chlamydia* species or cells stimulated with isolated chlamydial proteins can produce cytokines [40-43], but so far no comparative study has been conducted.

Although more and more data have been accumulating to better define the relationship between *Chlamydia* spp. and immune cells [6], there are no exhaustive details available about a significant difference in specific chlamydial species interaction with human monocytes.

## Conclusions

The present study outlines slight but constant and well defined delayed and/or lower reactive species response of human monocytes to CP in comparison with CT infection. This different response was observed in the early phase of infection and was related to such cellular defense mechanisms as ROS, NOS, and cytokines gene expression; consequently, CP killing activity by human monocytes was delayed by 24–28 hours when compared to CT. This feature is consistent with an early CP survival in monocytes *in vivo* infections, supporting the possibility that this bacterium can reach other more permissive cells.

## Competing interest

The authors declare they have no competing of interest.

## Authors’ contribution

AM, RF and RC conceived and designed the study; AM, CB, RF, CC, MD, PN, CF performed the experiments; AM, CC and CF analyzed the data; AM, CF, RC wrote the paper; all authors read and approved the final manuscript.
